# Modulation of accumbens dopamine by MCH neurons during learning and consummatory behavior

**DOI:** 10.1038/s41386-026-02351-z

**Published:** 2026-01-19

**Authors:** Liam E. Potter, Brandon A. Toth, Jayeeta Manna, Lorelei Baron, Hannah C. Lyons, Jack R. Evans, Christian R. Burgess

**Affiliations:** 1 Michigan Neuroscience Institute, University of Michigan, Ann Arbor, MI USA; 2 Department of Molecular and Integrative Physiology, University of Michigan, Ann Arbor, MI USA; 3 Neuroscience Graduate Program, University of Michigan, Ann Arbor, MI USA

**Keywords:** Reward, Hypothalamus

## Abstract

The formation of sensory cue-reward associations is essential for survival, but in the modern calorie-rich and advertising-intensive environment, such associations may become maladaptive - leading to negative health consequences such as obesity or diabetes. Recent research has demonstrated the importance of hypothalamic melanin-concentrating hormone (MCH)-expressing neurons in driving hedonically-motivated feeding and in forming these associations. The MCH system interacts with mesolimbic dopamine (DA) transmission, offering a potential mechanism for the effects of MCH neurons on hedonic feeding and associative conditioning. However, this interaction has not been fully characterized in vivo with modern approaches that offer high temporal and spatial resolution. We characterized MCH-DA interactions during feeding and food-motivated Pavlovian conditioning using in vivo fiber photometry in the lateral hypothalamus/zona incerta (LH/ZI) and nucleus accumbens (NAc). We found that MCH neuron activity and DA release in the medial-shell of the NAc (mNAcSh) were co-activated during consumption and in response to reward-predicting cues. During consumption, DA release preceded MCH activity, while responses to reward-predicting cues emerged in MCH neurons earlier than in the DA system. Lastly, gain and loss-of function of the MCH system could bidirectionally modulate DA release in the mNAcSh. These results indicate that physiological co-activation of the MCH and DA systems occurs during food-motivated learning, and demonstrate a capacity for bidirectional modulation of DA release in the mNAcSh by the MCH system.

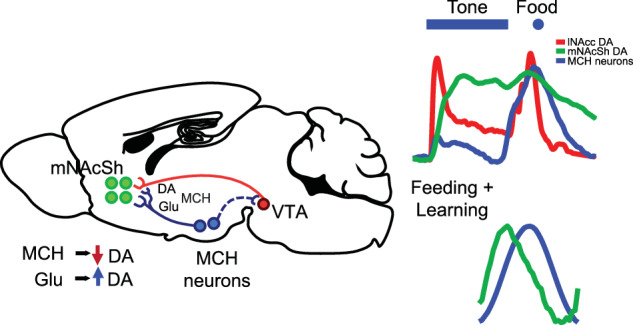

## Introduction

The formation of associations between environmental cues and food availability is critical to survival. The brain integrates external sensory cues with information about internal states to drive appetition and consumption - ensuring the necessary intake of nutrients. In our current environment, however, associations between ubiquitous environmental cues (e.g., advertisements) and hedonically rewarding foods can drive overconsumption, leading to metabolic dysregulation and obesity, with attendant health consequences and costs [1].

The importance of the mesolimbic dopamine (DA) system in learning reward-predicting sensory cues is well established [2]. Increased DA release in the nucleus accumbens (NAc) signals the availability of rewards and stimulates food seeking [3, 4]. The melanin-concentrating hormone (MCH) neuronal system, can increase food intake [5, 6] and is known to interact with the mesolimbic DA system [7, 8]. These neurons project widely throughout the brain, including to the ventral tegmental area (VTA) and NAc [9]. Interactions with reward systems have been postulated as a downstream mechanism underlying the effects of the MCH system on feeding and associative-learning [8, 10, 11]. MCH peptide injected into the NAc enhances food intake [12]. Optogenetic activation of MCH somas paired with food consumption enhances food intake [13], and/or increases preference for a paired food source while enhancing NAc DA tone [14]. Chemogenetic [15] or optogenetic activation of NAc-projecting MCH afferents [16] have similar behavioral effects. Studies have examined MCH-DA functional interactions, either in the context of consumption of drugs of abuse [7], in ex vivo preparations [17, 18], or in feeding [19]. However, these studies generally focused exclusively on MCH peptide, as opposed to MCH neurons, and/or employed temporally or spatially imprecise methods.

Here, we used fiber-photometry to characterize MCH activity and DA release dynamics within the NAc with high temporal precision during feeding and Pavlovian conditioning. We observed that DA dynamics and MCH neuron activity are moderately correlated and the relationship between these systems shifts during “behaviorally engaged” states. Cue-aligned DA responses emerge over the course of Pavlovian conditioning, while MCH responses to both cue and food-reward appear early in training and remain consistent. Gain and loss-of function of the MCH system could bidirectionally modulate DA release. These results indicate that co-activation of these systems occurs during food-motivated learning, and demonstrate a capacity for bidirectional modulation of DA release in the NAc by the MCH system. These interactions likely serve to promote food consumption and shape the trajectory of associative-learning.

## Materials and methods

### Mice

Protocols were approved by the University of Michigan’s Institutional Animal Care and Use Committee, and are in accordance with NIH guidelines for the use and care of Laboratory mice. MCH-Cre (Strain#:014099) mice and littermate controls were used in these experiments. PmchΔVglut2 (MCH-*vglut2-floxed*) mice, Pmch-iCre crossed with Slc17a6tm1Lowl/J mice (JAX stock # 012898) were also used (ref. PMID:17488640, PMID:39007235) in one experiment. These mice lack glutamate transmission from MCH neurons. All experiments were conducted between the hours of 11am-6pm. All mice were food-restricted to 85% of their free-feeding weight prior to and during experiments.

### Histology

We performed post-mortem histology on all animals to confirm fiber locations and construct expression (see Supplementary Fig. S1 for examples). In one figure we made use of an anterograde tracer experiment from the Allen Brain Connectivity Atlas [20, 21]. Eight images from the atlas were processed to remove background and thresholded prior to being quantified. We also quantified striatal MCH-ChrimsonR+ terminals in mice we generated (see optogenetics experiments for details on how mice were generated). 1–7 histology images from each of 3 mice were processed and quantified as above.

### In vivo fiber photometry

Photometry surgery and analysis were performed as in ref. [22]; (though see Supplementary methods). The fluorescent sensors dLight1.1 (green) and GRAB-rDA (red) were used to measure dopamine dynamics. For each recording session, the signal was converted to ΔF/F ((F – F_0_)/F_0_); where F_0_ was calculated as the 10th percentile of the entire fluorescence trace) and subsequently normalized as a z-scored ΔF/F. In some blue/green-light experiments we collected 405 nm (GCaMP isosbestic point) fluorescence to visualize motion artifacts (see Supplementary Fig. S7 for examples). For additional information on quantifications, see Supplementary Fig. S4.

### Feeding experiments

**Fig. 1:**
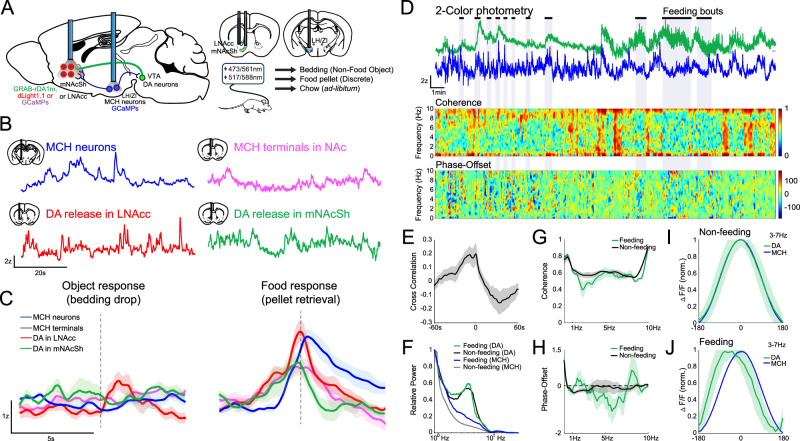
Fiber photometry recordings of DA release and MCH neuron activity during discrete and ad-libitum feeding. **A** Schematic of experimental recordings. **B** Example traces from MCH somas, MCH terminals in the mNAcSh, DA release dynamics in the lNAcc and mNAcSh. **C** Example responses to pellet retrieval or control (bedding) availability (i.e., drop into cage). **D** Simultaneous mNAcSh DA release dynamics (green) and MCH activity (blue) traces and feeding epochs during free-feeding. Coherogram and phase-offset relationship of 0.1–10 Hz DA and MCH oscillations across the same recording. **E** Mean cross-correlation between MCH and DA dynamics. **F** Example Fast-Fourier Transform spectrum for 0.1–10 Hz DA and MCH signals. **G**, **H** Coherence and phase-offset from 0.1 to 10 Hz during feeding and non-feeding epochs (*n* = 4). **I**, **J** Across-animals (*n* = 4) phase-offset relationship in the 3–7 Hz band during non-feeding and feeding epochs. See Supplementary Fig. S3E for quantification.

In ‘free feeding’ experiments, photometry and video recordings were initiated, and mice were placed in a fresh cage. Several pieces of regular chow were placed into one corner of the cage after ~30 s, this period was included in the main figure analysis (Fig. 1D–J) but excluded in the corresponding supplemental figure (Supplementary Fig. S3A–D). Mice were then allowed to interact with the food for 20–30 min. For subsequent analyses, photometry data was synchronized with the video recording, and a human scorer noted the times during which the mouse consumed the chow.

### Pavlovian conditioning

Mice had two 30 min sessions to habituate to the recording chamber, in-cage FED3 [23], and optic cable and were allowed to freely receive pellets. Mice were trained across 3–11 subsequent sessions to associate a 1 kHz, 5s-duration tone with pellet delivery. ~40 tones were played per session, with pellet delivery occurring 5 s after tone onset, and a variable 60–90 s ITI. Both the start of the tone and the moment of pellet retrieval were timestamped via TTL pulses.

### Gain and loss of function experiments

For chemo-inhibition experiments, MCH-Cre mice were injected with AAV5-hSyn-DIO-HM4Di(Gi)-mcherry and dLight1.1 (mNAcSh). Prior to the start of 1-2 Pavlovian conditioning runs, mice were habituated to I.P. injections of saline. Mice were pretreated with either vehicle or 3 mg/kg CNO 30 min. prior to running in the Pavlovian task.

For optogenetics experiments, MCH-Cre mice were injected with AAV5-Syn-FLEX-rc[ChrimsonR-tdTomato] and dLight1.1 (mNAcSh), and implanted with mNAcSh fibers. In a subset of trials (70%:30% stim:no-stim) either the tone, the pellet, or both (~10% of stim trials) were paired with optogenetic stimulation (5s-duration, 20 Hz, 10 ms pulse-width, 625 nm).

### Pharmacology

For MCHR1 inhibition experiments, 45 min. prior to the start of each run mice were treated with the MCHR1 antagonist SNAP-94847 (25 mg/kg, I.P.). Mice were habituated to I.P. injections of saline.

### 2-color photometry analyses

Analysis was performed as in ref. [24], using MATLAB scripts adapted from their work. Briefly, after normal signal processing, synchronously recorded GRAB-rDA1m and GCaMP were analyzed for cross-correlation, coherence and phase-offset. Coherograms used a multi-taper estimation with the chronux function cohgramc. To generate the phase-offset curves, we likewise adapted the analysis in ref. [24] – briefly, the signals were processed with a Butterworth (bandpass) filter and the phase angle of the Hilbert function was extracted. Fluorescence amplitudes were extracted and averaged in 10 degree bins from −180:+180 degrees. The curves were normalized across the full oscillatory cycle and superimposed.

### Statistical analysis

Statistical analyses were performed using Prism 9.0 or Matlab software. Data presented met the assumptions of the statistical test employed. N numbers represent final numbers of healthy/validated mice, except in the case of one mouse where histology was unavailable.

## Results

### MCH and mesolimbic DA systems respond to discrete food-rewards, and are correlated during feeding

Initially, cohorts of mice expressing the DA sensor dLight1.1, with recording fibers implanted in either the lateral core of the nucleus accumbens (lNAcc) or its medial shell region (mNAcSh) – where we have found MCH+ fiber innervation is densest (see ref. [9] and Supplementary Fig. S2*)* – were used to observe DA release dynamics (see Supplementary Fig. S1A for an overview of cohorts/constructs). *pMCH-Cre* mice expressing GCaMP, with recording fibers implanted in either the LH, or both the mNAcSh and the LH, were used to observe somatic and axonal calcium-dynamics (Fig. 1A). In either case, each mouse expressed only one fluorescent sensor at the recording fiber. We observed spontaneous dynamics in freely behaving mice (Fig. 1B). Both the MCH and DA systems were transiently activated by consumption of discrete food rewards (Fig. 1C) but were only minimally activated when a similarly-sized non-food object (a piece of bedding) was dropped into their cage.

In a second experiment, we made use of mice expressing both a red fluorescent sensor for DA (GRAB-rDA1m) with a recording fiber in the mNAcSh, and a green sensor (GCaMPs) for MCH neuron activity recorded from the LH. Food-restricted mice were placed in a fresh cage while recording MCH/DA dynamics simultaneously, and videography was used to determine when mice were engaged in food consumption (Fig. 1C, D). Cross-correlation analysis between MCH activity and DA release dynamics showed a moderate level of correlation over a range of several seconds (maximum correlation mean: 0.250 ± 0.072) (Fig. 1E). We then refined our analysis to focus on a physiologically relevant range of oscillatory frequencies (i.e. 0.1–10 Hz). As has been reported elsewhere [24], a prominent band of DA activity was observed around 4 Hz (Fig. 1F). In comparison, MCH neurons did not show a clear peak of rhythmic activity (Fig. 1F). We found that MCH/DA coherence was generally moderate (~0.6) in both feeding and non-feeding conditions (Fig. 1G), though higher (~0.8) at low frequencies (<1 Hz). There was little offset between the two signals in non-feeding conditions (Fig. 1H, I); however, within the 3–7 Hz band where DA showed rhythmic activity, DA release was observed to shift forward its phase relative to MCH activity during feeding (Fig. 1H, J, Supplementary Fig. S1E). This suggests that the two systems may be differentially driven during food consumption compared to non-consumption epochs. Excluding the period prior to food introduction from the analysis had little effect on the relationship (Supplementary Fig. S3A–D).

### The MCH system responds to food-predictive cues early in Pavlovian conditioning

**Fig. 2:**
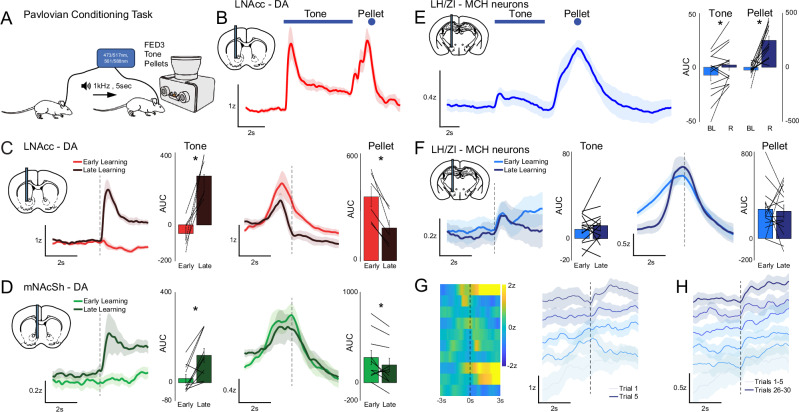
Changes in DA release dynamics and MCH neuron activity over Pavlovian conditioning. **A** Schematic of Pavlovian conditioning. **B** lNAcc DA release dynamics (*n* = 6) showing response peaks to both the onset of the cue and the food-reward (pellet). **C** lNAcc DA release (n = 6) showing cue responses emerge over learning (inset: AUC of baseline-corrected Response Early vs. Late (Q1 vs. Q5), two-tailed paired *t*-test **p = 0.0043, *t* = −4.945, df = 5). Pellet responses diminish over learning (Early vs. Late, **p = 0.0025, *t* = 5.623, df = 5). **D** mNAcSh DA release (*n* = 10) showing cue responses emerge over learning (Early vs. Late, two-tailed paired *t*-test **p = 0.0024, *t* = −4.1737, df = 9). Pellet responses diminish over learning (Early vs. Late, *p = 0.0403, *t* = 2.3933, df = 9). **E** MCH neuron activity (*n* = 14) demonstrating responses to the cue and pellet (AUC of baseline vs. AUC of Response, all trials, tone *p = 0.0273, *t* = −2.4856, df = 13, pellet ***p = 0.0001, *t* = −5.3403, df = 13). **F** MCH (*n* = 14) cue and pellet responses do not change over learning once initially established (Early vs. Late, n.s.). **G** Heatmap of peri-event (tone) MCH dynamics over the first 10 cue-reward pairings for a representative individual (left), and group mean traces (*n* = 6), over the first 5 cue-reward pairings (right). **H** Responses over the first 30 cue-reward pairings, after binning together every 5 consecutive trials (right). In responsive animals, MCH neuron tone-responses emerge rapidly, but are smaller when compared to MCH pellet responses or DA tone responses.

To have more control over the timing of feeding and to look at responses to food-predicting sensory cues, we shifted our focus to Pavlovian conditioning. Mice underwent between 3 and 11 days (7.2 ± 0.63 runs/mouse) of conditioning with a daily run lasting 31–60 min (45.2 ± 2.3 min/run). On average, mice took 37.5 ± 2.5 (Range: 24–55) food pellets per run. We initially observed changes in lNAcc DA release dynamics over the course of Pavlovian conditioning (Fig. 2B). At early stages of conditioning, we observed a large phasic release peaking immediately prior to initial consumption of the food pellet, but no response to the auditory cue (Fig. 2C). After repeated pairings of the auditory cue with the pellet (44.0 ± 4.3 trials/quintile, Range: 34–60*)*, a strong phasic DA response to the cue developed, with the pellet response diminishing in magnitude. Over the course of a run in late learning, DA responses to the pellet but not the cue were attenuated – indicating some habituation to novelty or enhanced expectancy of the reward (Supplementary Fig. S5B). These changes in lNAcc DA release dynamics are in line with classical reward-prediction error (RPE) [2]. In the mNAcSh, which is most strongly innervated by the MCH system, DA responses shifted from the pellet toward the cue in late-learning (Mean 46.9 ± 5.9 trials/quintile, Range: 23.2–87*)* (Fig. 2D). DA release dynamics in the mNAcSh were qualitatively like those in the lNAcc but peaked during consumption rather than prior to consumption. DA release in the mNAcSh elicited by the cue in late learning tended to ‘ramp’ toward a plateau which was maintained through consumption of the pellet, as opposed to hitting a sharp peak followed by a rapid decline. Within a run, there was less decline in DA release in later vs. earlier trials (Supplementary Fig. S5C).

MCH neurons were also activated strongly when mice approached and consumed the food pellets (Fig. 2E). Unlike DA release, there was a clearly defined but small (relative to the pellet response) response to the cue appearing very early in conditioning (Fig. 2E–H). This cue response was observable in a subset of animals (6/14) within the first 5 cue-reward pairings (Fig. 2G) and was consistent in shape and magnitude after that (Fig. 2H). There was little change in magnitude of the cue response from the first quintile of trials to the last (Fig. 2F) (59.4 ± 4.1 trials/quintile, Range: 27.8–77.2), apart from a more consistent ‘ramping’ of activity toward the pellet response early on. There was no significant attenuation of the pellet response in late vs. early learning. We also confirmed that mNAcSh projecting MCH neurons are indeed activated during our Pavlovian conditioning task by recording from terminals in that region (Supplementary Fig. S5A). Taken together, we can speculate that although the MCH system is activated by both the predictive cue and food reward across essentially all stages of conditioning, it does not function in an ‘RPE-like’ predictive manner – although there may also be a novelty component involved in MCH activation [25, 26]. Co-activation of the MCH and DA systems across conditioning may shape behavioral responses, promoting task engagement and/or reward consumption and perhaps shaping activity in the NAc in both a pre- and post- synaptic manner by modulating DA release and interacting with postsynaptic medium-spiny neurons (MSNs) [11, 19, 27, 28].

We performed within-animal simultaneous 2-color photometry of MCH activity and DA dynamics (Supplementary Fig. S6A). We confirmed that, as suggested by our asynchronous observations, the MCH system is activated consistently by food-predictive cues very early in learning, prior to the emergence of DA responses to cue. This is clearest when observing responses during the first 30 trials (Supplementary Fig. S6B–D). After roughly 50 trials in our paradigm, clear DA responses to cue emerged and continued to increase in magnitude, whereas MCH responses remained relatively small compared to pellet responses or DA responses (Supplementary Fig. S6D). All subsequent experiments targeting the NAc focused on the mNAcSh region, where MCH innervation is densest (Supplementary Fig. S2).

### Correlation, coherence and phase relationships between MCH and DA systems during Pavlovian conditioning resemble feeding

**Fig. 3:**
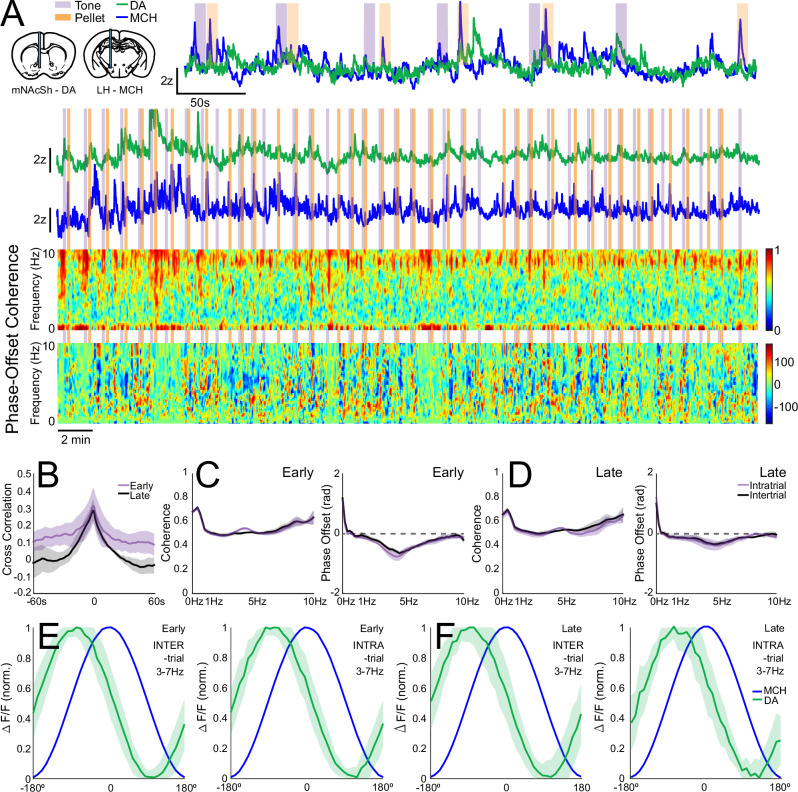
Coherence and phase-relationship between mNAcSh DA and LH/ZI MCH neuron dynamics across Pavlovian conditioning. **A** (Top) Example simultaneous DA release and MCH activity during Pavlovian conditioning at 2 different timescales. Coherogram (middle, relative power) and phase-offset relationship (bottom, degrees) of 0.1–10 Hz DA and MCH oscillations across the same recording. **B** Cross-correlation between MCH/DA dynamics. Coherence between DA and MCH dynamics from 0.1 to 10 Hz during cue/reward presentations or ITI, in early (*n* = 3; **C**, Left) and late (*n* = 3; **D**, Left) learning. Phase-offset relationship from 0.1-10 Hz during cue/reward presentations or ITI, in early (**C**) and late (**D**) learning. Phase-offset relationship in the 3–7 Hz band during ITI or cue/reward presentations, in early (**E**) and late (**F**) learning.

We next examined the degree of coherence and the phase-relationship between activity in the MCH/DA systems over the course of Pavlovian learning. We compared activity within the intertrial-interval (ITI) to activity within a trial (i.e., during the cue-reward period; Fig. 3A). We observed a moderate degree of cross-correlation between MCH activity and DA release dynamics over a range of several seconds, with little change over learning (early learning maximum correlation mean = 0.292 ± 0.066, late learning mean = 0.326 ± 0.109). Similarly, we observed moderate coherence (~0.5) between the 2 signals in the 0.1–10 Hz range during trials and in the ITI period (Fig. 3C, D). Unlike in the free-feeding paradigm, there was a clear phase-offset between the 2 signals in the 3-7 Hz band whether we looked within the trial period or in the ITI (Fig. 3D, E, Supplementary Fig. S3F, G). Activity during the Pavlovian conditioning paradigm therefore more closely resembled the feeding condition in this regard. This pattern of activity may therefore be reflective of task engagement, as opposed to a correlation with feeding per se.

### Inhibition of the MCH system does not block the emergence of mNAcSh DA responses to cue in Pavlovian conditioning

**Fig. 4:**
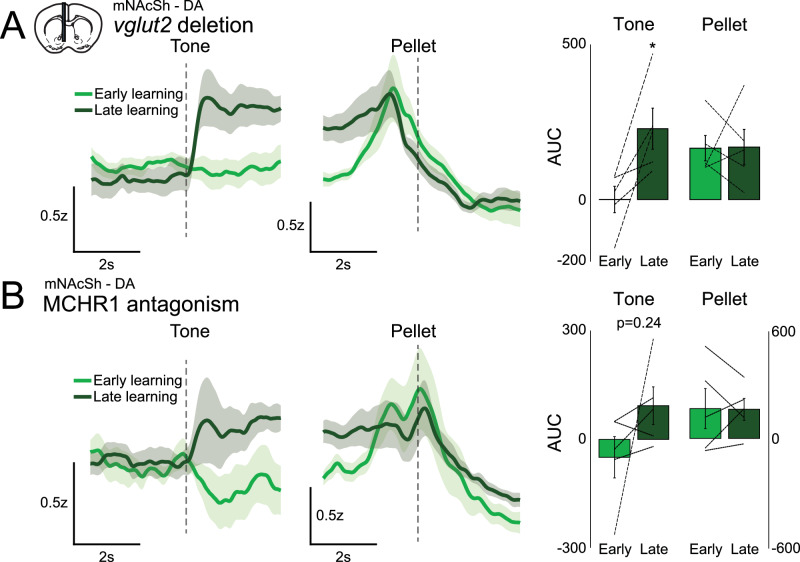
Effects of chronic MCH loss-of-function on mNAcSh DA release. **A** Cue-aligned or pellet-aligned DA responses in MCH-vglut2-floxed mice during learning (AUC of Response – Baseline, Early vs. Late, *n* = 5, tone two-tailed paired *t*-test *p = 0.0298, *t* = −3.3052, df = 4, pellet n.s.). **B** Cue-aligned or pellet-aligned DA responses in MCHR1-inhibited mice during learning (Early vs. Late, *n* = 5, tone two-tailed paired *t*-test *p* = 0.2356, *t* = −1.3947, df = 4, pellet).

To constitutively block glutamate release from MCH neurons, we made use of *MCH-vglut2-floxed* mice. Blockade of glutamate release had little observable effect on the emergence of DA responses to auditory cues over learning (Fig. 4A). This suggests that glutamate release from MCH neurons is not critical for this type of learning, although we cannot exclude compensatory changes in this model. To block the effects of the MCH peptide, we pretreated experimental mice daily with the MCH-receptor 1 (MCHR1) antagonist SNAP-94847 (25 mg/kg I.P., 45 min prior to each recording/training day). Both cohorts received a minimum of 5 days of training (max was 8 days). Pharmacological blockade of the MCHR1 did not completely prevent the emergence of DA responses to cue (Fig. 4B); however, it appeared to reduce task-engagement, which likely contributed to a delayed emergence of cue-response (Supplementary Fig. S8A, C). Because we did not run a simultaneous group of control mice, we cannot exclude that there may be changes in the rate of learning with loss of MCH neuron or peptide function. Also of note in these groups, we do not see the same decrement in the magnitude of pellet responses over learning as we initially observed (Fig. 2D) – this may be attributed to lower overall trial counts in the treatment groups or may be an effect of the manipulations.

### Acute manipulations of the MCH system demonstrate bidirectional modulation of DA release in the mNAcSh

**Fig. 5:**
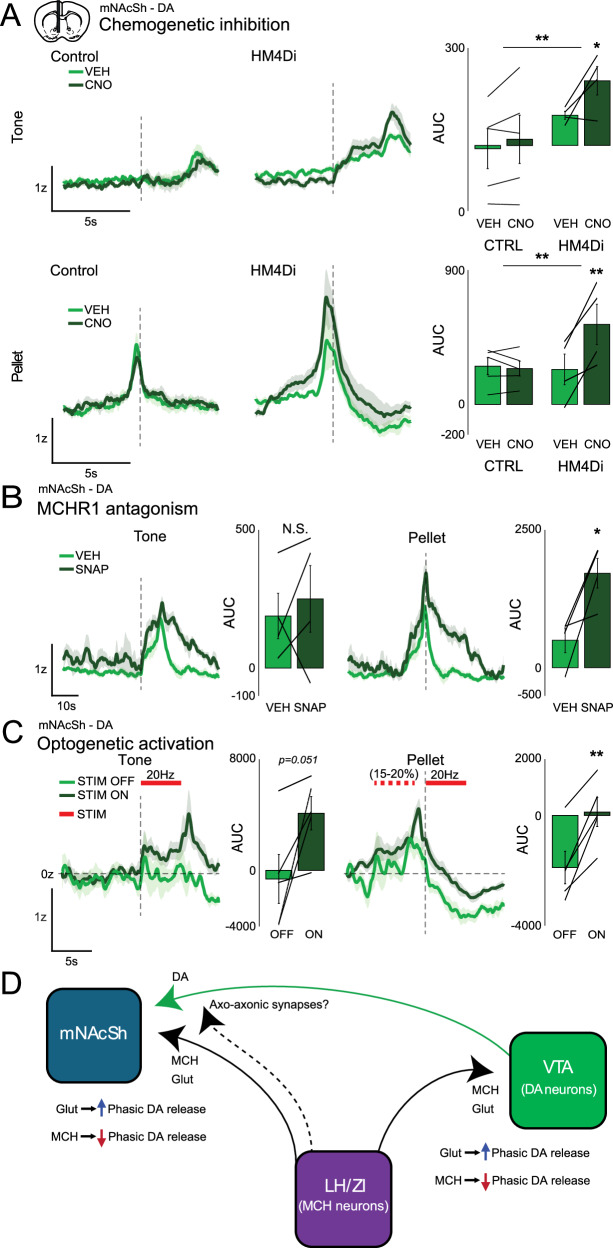
MCH neurons bidirectionally modulate DA release. **A** Cue-aligned or pellet-aligned DA responses in VEH- or CNO-treated control (CTRL) or MCH-HM4Di animals during late-learning (*n* = 5/4; 2-Way RM-ANOVA VEH vs CNO-treated, Control vs MCH-HM4Di group, Control *n* = 5, HM4Di *n* = 4. Tone: Significant overall effect of treatment *p = 0.0162, treatment × genotype *p* = 0.1105, Sidak post-hoc, HM4Di-VEH vs. HM4Di-CNO *p = 0.049. All other comparisons n.s.; Pellet: VEH vs CNO-treated, Control vs MCH-HM4Di group, Control *n* = 5, HM4Di *n* = 4. Significant overall effect of treatment **p = 0.0045, treatment × genotype **p = 0.0026, Sidak post-hoc, HM4Di-VEH vs. HM4Di-CNO **p = 0.0026. All other comparisons n.s.). **B** Cue-aligned or pellet-aligned DA responses in animals treated with VEH or SNAP-94847 during late-learning (AUC of Response – Baseline, VEH vs. SNAP-treated, *n* = 4, tone two-tailed paired *t*-test n.s., pellet *p = 0.0423, *t* = −3.3406, df = 3). **C** Cue-aligned or pellet-aligned DA responses in MCH-ChrimsonR mice, opto-paired (Opto) and unpaired trials (No Opto). Period of opto-excitation indicated in red (AUC over stim window, Opto unpaired (OFF) vs. paired (ON) trials, *n* = 5, tone two-tailed paired *t*-test *p* = 0.05123, *t* = −2.7527, pellet **p = 0.00365, *t* = −6.1013, df = 4). **D** Schematic of proposed interactions between MCH and DA systems.

Although the MCH system is not necessary for the emergence of normal DA dynamics in the mNAcSh over the course of Pavlovian conditioning, this does not preclude the possibility that the MCH system may modulate DA release in this context. In MCH-HM4Di mice (an inhibitory chemogenetic receptor), but not control mice, pretreatment with CNO led to a significant enhancement of DA release, particularly noticeable during the pellet response (Fig. 5A), compared to vehicle in the same animals. Likewise, when well-trained WT mice were pretreated with the MCHR1 antagonist, DA responses were significantly enhanced compared to vehicle in the same animals (Fig. 5B). These results are likely explained by ongoing (tonic) suppression of DA release by the MCH peptide. As these approaches are systemic it is possible that the effects are mediated at the level of the VTA or NAc.

Enhancement of DA release appeared greater in the SNAP-94847 treated mice compared to chemo-inhibition of MCH neurons. We therefore hypothesized that glutamate release from MCH neurons might enhance DA transmission. Chemogenetic inhibition of MCH neurons is less effective at enhancing DA release versus MCHR1 blockade due to the opposing effects of inhibiting glutamate release from MCH neurons versus reducing MCH peptide release. To test this, we optogenetically stimulated MCH terminals in the mNAcSh in mice expressing the red-shifted excitatory opsin ChrimsonR in MCH neurons, and dLight1.1 in the mNAcSh. Once trained, we paired brief stimulation of MCH terminals with either the cue, the pellet, or both, with non-stimulated trials interspersed. We found that activation paired with either the tone or the pellet enhanced DA release compared to non-stimulated trials (Fig. 5C). Although it is possible MCH/MCHR1 signaling may play a role in this phasic enhancement, the relatively brief nature of the activation implies that it is more likely mediated by glutamate release.

## Discussion

Several studies on how the MCH system acts to influence hedonic feeding or food-motivated learning have pointed to an interaction between the MCH and the mesolimbic DA systems [11, 12, 14, 16, 17, 19]. Here, we used recording and manipulation approaches to demonstrate the relationship between MCH activity and DA release with high levels of temporal and spatial precision, providing evidence of functional co-activation during feeding and learning, and of a causal interaction between MCH neuron activity and DA release in the mNAcSh.

We found that prior to any learning, MCH neurons are activated and DA release occurs in response to food-rewards. As in Subramanian et al. [10], we found that pairing an auditory cue with food delivery led to the emergence of MCH responses to the cue, indicating that they may signal reward expectancy. However, we did not see significant continued increases in magnitude of the MCH neuron response beyond the first day of learning. Rather, once cue-responses emerged, which occurred very early in training, they remained consistent over the course of training. This contrasts with the changes we observed in NAc DA release dynamics, which appeared later in training and continued to evolve and increase over the multi-day learning period. The early appearance of MCH cue-responses could suggest that MCH activity may shape emerging DA responses from very early in learning. MCH neuron activity has also been associated with novelty. Previous work [25, 26] shows that MCH neurons are activated by novel-object presentation. Therefore, early cue-responses could signal novelty, except that we did not see attenuation of MCH cue responses across learning or within a run.

MCH and DA systems were co-activated during reward and cue presentation in the Pavlovian paradigm. Indeed, there is a moderate (~0.5 at >1 Hz) to high (~0.7 at <1 Hz) degree of correlation and coherence between these two systems across a range of frequencies and behavioral states. This strengthens the argument that the systems are functionally coupled, and serve related behavioral functions, though these data do not identify the nature of this coupling. Based upon the apparent offset of DA and MCH dynamics, MCH may not be a major driver of DA rhythms – but may modulate it over a range of timescales. Additional studies may need to be conducted that employ better kinetically-matched sensors, longer recording periods, and more varied contexts (e.g., sated vs. food-deprived) to fully appreciate how these two systems are coupled.

We hypothesized that chronic loss of MCH neuronal function may slow the acquisition of DA responses to cues during learning. Neither blockade of glutamatergic signaling or MCH signaling abolished emergence of cue responses over learning. Repeated MCHR1-antagonist treatment may, however, reduce task engagement, leading to slightly slower cue-response learning. These findings suggest that the MCH system is not required for the normal acquisition of phasic DA responses during Pavlovian conditioning, but instead may serve to augment food-seeking and drive task engagement. Systemic pharmacological antagonism of MCHR1 may also have off-target effects on other receptors and brain regions, however, both acute pharmacological inhibition of MCHR1 signaling and chemogenetic inhibition of MCH neurons produced increases in DA release. These data support previous work suggesting that MCH peptide has an inhibitory effect on DA signaling [26, 27], with loss of peptide function therefore producing disinhibition. It is not clear from these experiments if this effect is primarily at the VTA or within the NAc. We also found that brief, acute activation of MCH terminals in the mNAcSh increased DA release, suggesting that MCH neuronal activity is capable of enhancing DA release. Such brief opto-activation favors fast-neurotransmitter release, though we cannot exclude possible co-release of MCH peptide. These observations may be reconciled by a proposed circuit in which MCH peptide/MCHR1 act tonically via G_i/o_ at VTA dopamine neurons to suppress DA tone in the NAc and glutamate signaling in the NAc (and potentially directly at VTA-DA, via backpropagation and axon collaterals) induces DA release. This proposed circuit (see Fig. 5D, Supplementary Fig. S9*)* thus fits both with previous experiments [17, 18, 29, 30] which found that VTA-DA neurons were inhibited by MCH, in that *pMCH* deletion or MCR1 blockade led to a hyperdopaminergic state – and with experiments which found stimulation of MCH neurons increased NAc DA tone [14, 17]. Notably, Spencer et al. [18] observed that most VTA-DA neurons express MCHR1 and superfusion of MCH suppressed firing in VTA-DA neurons in slice. Also of note, Schneeberger et al. [31] found that the effects of MCH neuron ablation on sucrose preference were primarily glutamate-mediated. That said, as we do not *directly* observe an MCH-induced reduction in DA release (versus a disinhibitory effect of blocking MCHR1) in any experiment, an alternative model of MCH-DA interactions based on an “inverted-U” shaped (i.e., homeostatic) dose-response cannot be ruled out, as observed by Noble et al. [32–34] in a similar context [32]. Here, we add to our understanding of the MCH system and its role in modulating mesolimbic DA transmission in the context of Pavlovian learning and food consumption, while acknowledging that additional work will be required to fully elucidate these mechanisms. We also do not address the effects of phasic MCH release on DA release, or the question of post-synaptic effects of MCH and DA in the NAc, though receptors for both transmitters are present on most MSNs [19, 27, 28]. Ultimately, MCH neurons are only one of many modulatory influences on reward systems that shape food-seeking and consummatory behavior.

## Supplementary information

Supplemental figures and methods

Table S1

Table S2

## Data Availability

Original data is available from the Burgess Lab upon request.
