# Supplementary material for: Modulation of accumbens dopamine by MCH neurons during learning and consummatory behavior
Source: Neuropsychopharmacology. 2026 Jan 19;51(7):1217–25. doi: 10.1038/s41386-026-02351-z (PMC13212990; doi:10.1038/s41386-026-02351-z)
Supplement: Supplementary file 1 — Supplemental figures and methods [file 41386_2026_2351_MOESM1_ESM.docx]

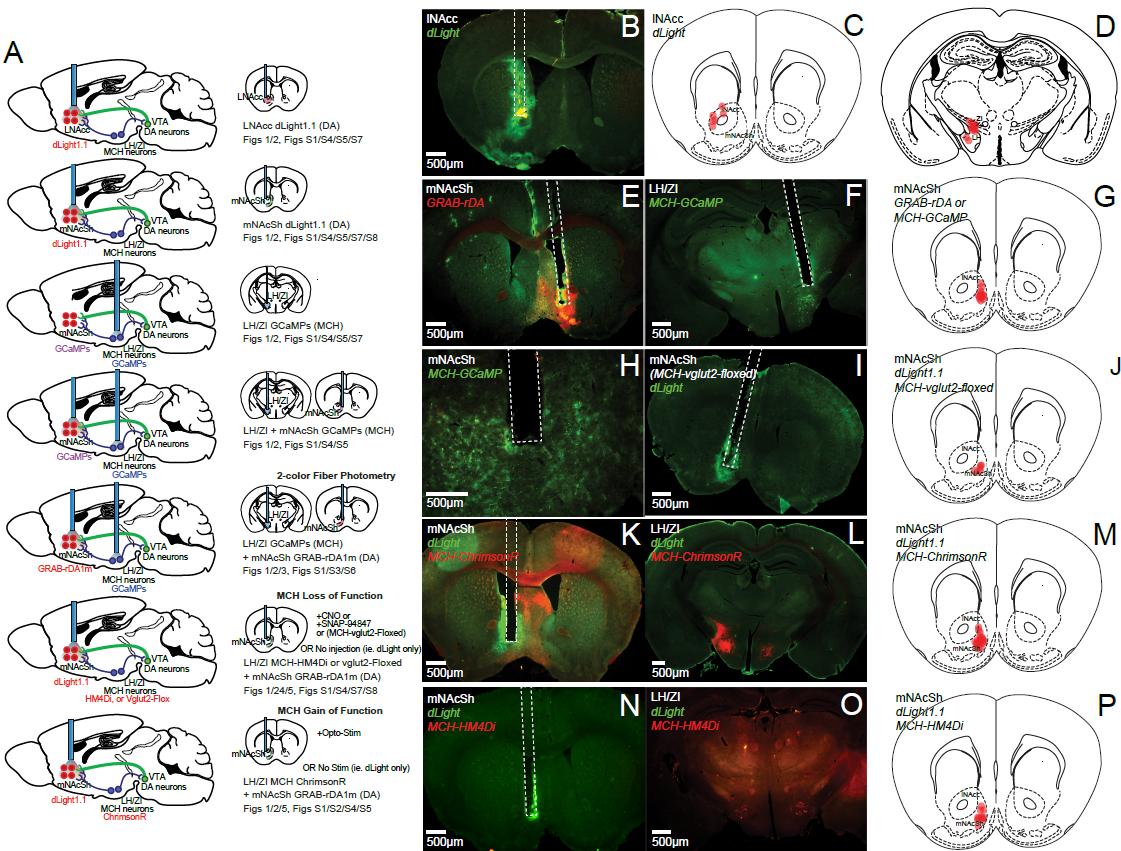


**Figure S1. Overview of experiments and representative histology and fiber locations.**

A) Schematic diagrams of various sensors, actuators, and fiber-locations. *Note these schematics are not intended to be seen as mutually exclusive, ie. in some experiments groups were comprised of mice with unused actuators/sensors/fibers which were used elsewhere. B) Representative photomicrograph of dLight1.1 expression in lNAcc. (Fiber tract in hatched-white lines). C) Observed fiber-tip locations in experimental animals (lNAcc, dLight1.1). AP coordinate +1.1mm from bregma, may vary in individuals by approx. +/-0.2mm. D) Observed fiber-tip locations in experimental animals (LH/ZI, MCH-GCaMP). AP coordinate +1.7mm from bregma, may vary in individuals by approx. +/-0.2mm. E) Representative photomicrograph of GRAB-rDA1m expression in mNAcSh. F) Representative photomicrograph of MCH-GCaMP expression in LH/ZI. G) Observed fiber-tip locations in experimental animals (mNAcSh, GRAB-rDA1m). AP coordinate +1.1 mm from bregma, may vary in individuals by approx. +/-0.2mm. H) Close-up (20x objective) representative photomicrograph of MCH-GCaMP expression in mNAcSh. I) Representative photomicrograph of dLight1.1 expression in mNAcSh. J) Observed fiber-tip locations in experimental animals (mNAcSh, dLight1.1, MCH-vglut2-floxed). AP coordinate +1.1 mm from bregma, may vary in individuals by approx. +/-0.2mm. K) Representative photomicrograph of dLight1.1 expression in mNAcSh in MCH-ChrimsonR co-expressing mice. L) Representative photomicrograph of MCH-ChrimsonR expression in LH/ZI. M) Observed fiber-tip locations in experimental animals (mNAcSh, dLight1.1, MCH-ChrimsonR co-expressing mice). AP coordinate +1.1 mm from bregma, may vary in individuals by approx. +/-0.2mm. N) Representative photomicrograph of dLight1.1 expression in mNAcSh in MCH-HM4Di co-expressing mice. O) Representative photomicrograph of MCH-HM4Di expression in LH/ZI. P) Observed fiber-tip locations in experimental animals (mNAcSh, dLight1.1, MCH-HM4Di co-expressing mice). AP coordinate +1.1 mm from bregma, may vary in individuals by approx. +/-0.2mm.

**
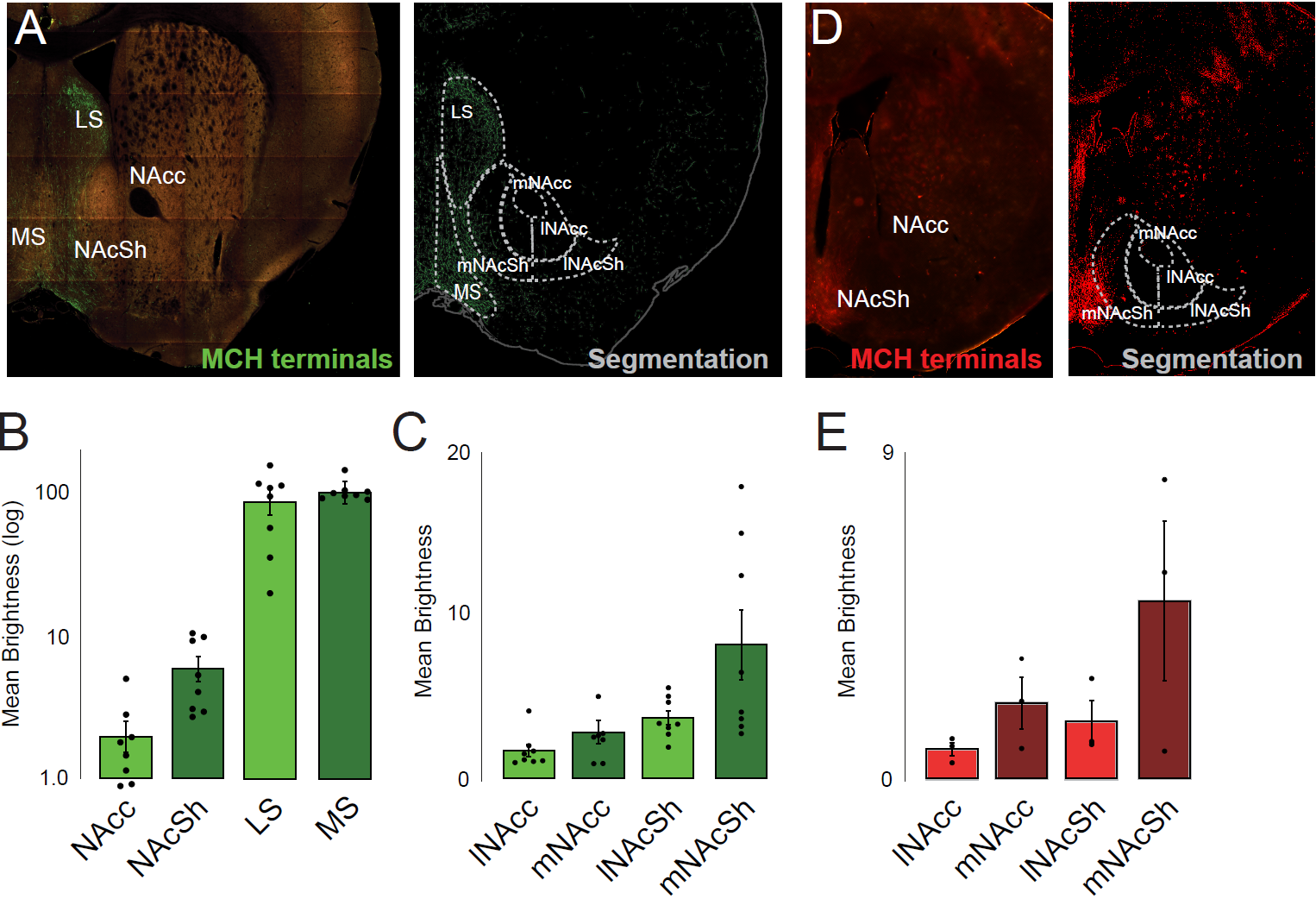
**

**Figure S2. Anatomical distribution of MCH+ fibers in the NAc.**

A) Representative photomicrograph from the Allen Brain Connectivity Atlas, anterograde-tracing experiment in pMCH-Cre mice (injected in the LH). Segmentation to delineate medial and lateral subregions. Image and dataset from Allen Mouse Brain Connectivity Atlas, <https://connectivity.brain-map.org/projection/experiment/113444277/> Image credit: Allen Institute for Brain Science. <https://connectivity.brain-map.org/projection/experiment/siv/113444277?imageId=113444467&imageType=TWO_PHOTON,SEGMENTATION&initImage=TWO_PHOTON&x=19487&y=16325&z=3>. B) Quantification of fluorescence (MCH+ fibers) in NAc and septum. C) Quantification of fluorescence (MCH+ fibers) in subregions of the NAc. D) Representative photomicrograph from our lab, MCH-ChrimsonR expressing terminals in the NAc. Segmentation to delineate subregions. E) Quantification of fluorescence (MCH+ fibers) in subregions of the NAc.


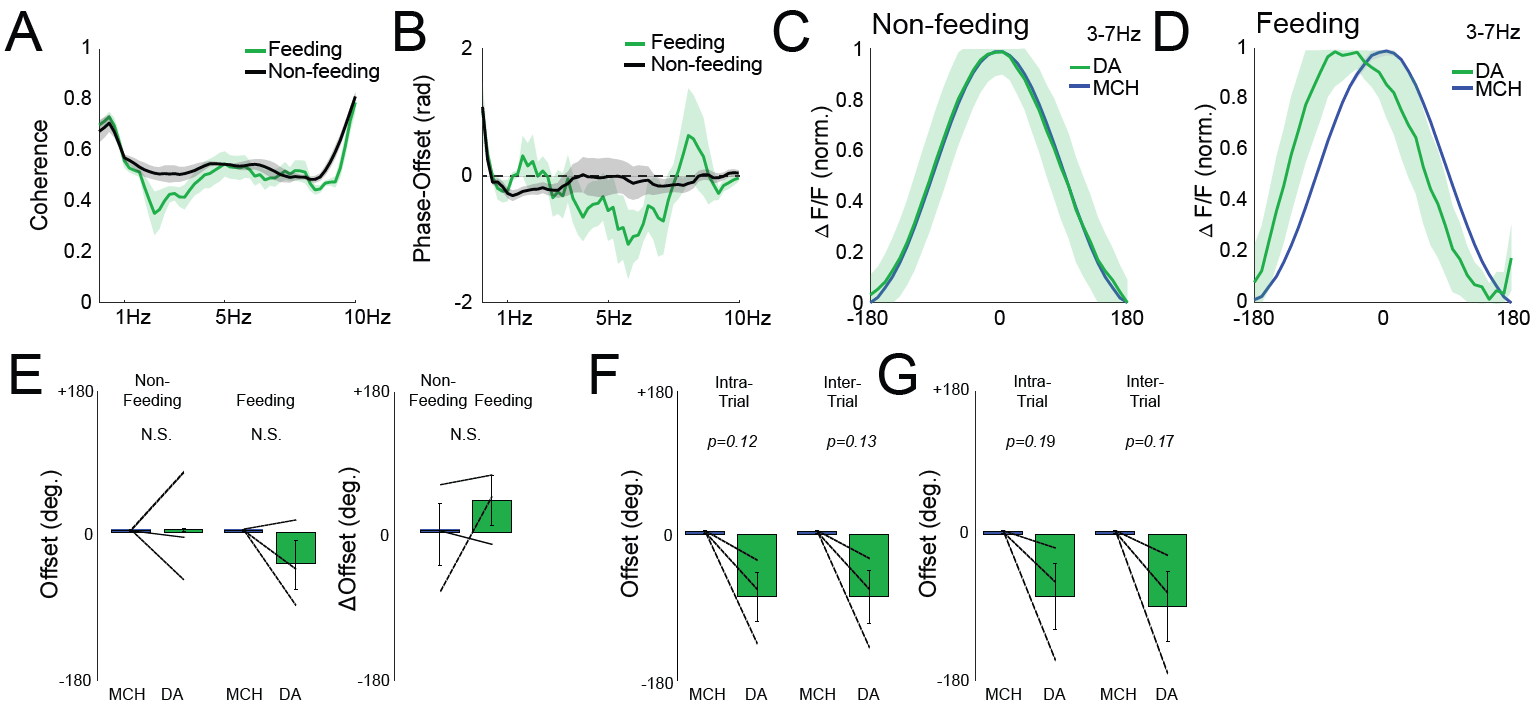


**Figure S3. Alternate time-binning for free-feeding experiment and quantification of phase-offset curves.**

A, B) Coherence and phase-offset from 0.1-10Hz during feeding and non-feeding epochs (n=3) after exclusion of the period prior to introduction of food into the cage. C, D) Across-animals (n=3) phase-offset relationship in the 3-7Hz band during non-feeding and feeding epochs after pre-food period excluded. Differences were negligible compared to original time-binning so offset curves were not quantified. E) Quantification of half-height phase shift (degrees) of DA relative to MCH in non-feeding (two-tailed paired t-test p=0.9974, t=0.00364, df=2) (left) and feeding (two-tailed paired t-test p=0.3043, t=1.370, df=2) conditions, and the relative shifts (DA-MCH) in each state compared (right) (two-tailed paired t-test p=0.4030, t=1.052, df=2). F) Quantification of half-height phase shift (degrees) of DA relative to MCH in intra-trial (two-tailed paired t-test p=0.1196, t=2.626, df=2) and inter-trial conditions (two-tailed paired t-test p=0.1264, t=2.538, df=2) (left) in early Pavlovian learning. G) Quantification of half-height phase shift (degrees) of DA relative to MCH in intra-trial  (two-tailed paired t-test p=0.1934, t=1.930, df=2) and inter-trial conditions (two-tailed paired t-test p=0.1669, t=2.130, df=2) (left) in late Pavlovian learning.


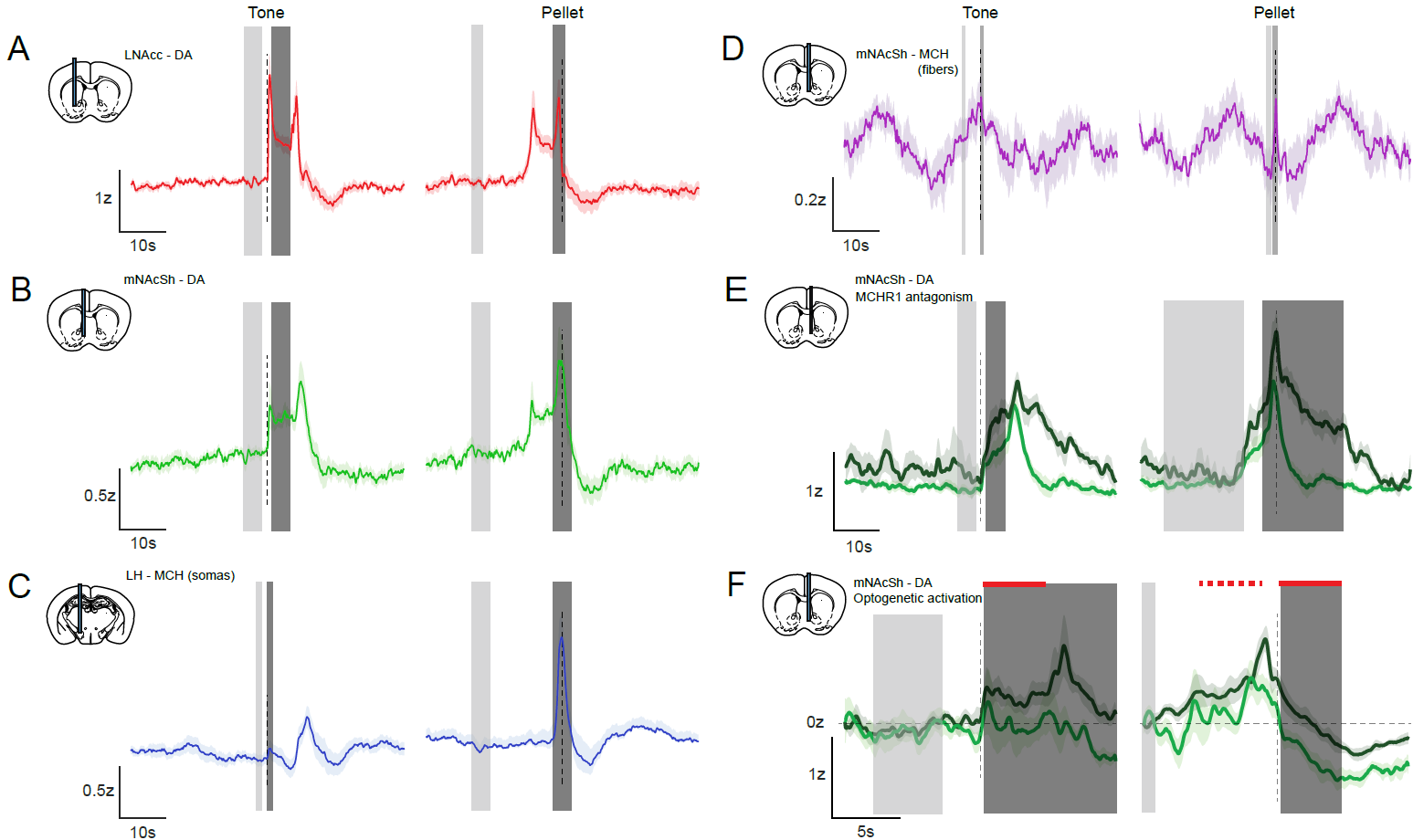


**Figure S4. Quantification windows for photometry.**

A) Quantification windows for lNAcc (dLight) photometry – tone- and pellet retrieval-locked responses. (*Note: different timescale here vs. main figures). Light grey – Baseline period, Dark grey – Response period. AUCs were calculated as AUC of response minus AUC of baseline. B) As above, for mNAcSh (dLight), including most LOF studies. C) As above, for LH/ZI (MCH-GCaMPs, somas). D) As above, for mNAcSh (MCH-GCaMPs, terminals). E) As above, for acute MCHR1 antagonism experiment. F) As above, for acute MCH-opto-activation experiment. Note: different timescale vs A-E. Red – Stim ON period.


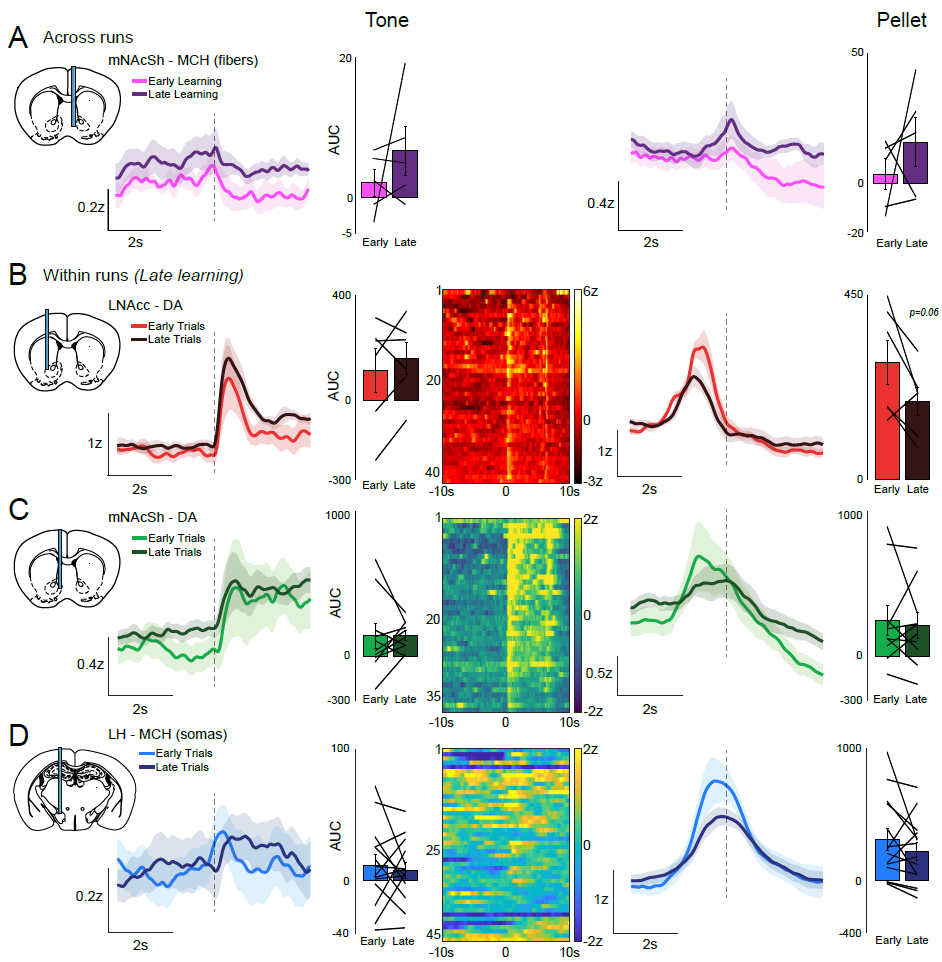


**Figure S5. MCH axon-terminal responses to food-reward and cue in the mNAcSh, and changes in DA release dynamics and MCH neuron activity over the course of a Pavlovian conditioning run.**

A) mNAcSh MCH axon-terminal dynamics (mean across animals, n=5) during early and late learning. Cue responses trended towards an increase but generally showed low signal-to-noise ratio as well as an unstable baseline (tone) (AUC of Response – Baseline, Early vs. Late, n=5, two-tailed paired t-test p=0.3742, t=-0.9993, df=4). Food-reward (pellet) responses increase over learning N.S. (Early vs. Late, n=5, paired t-test=0.3819, t=-0.9815, df=4). Although recordings from terminals are often more technically challenging to achieve and are noisier (vs. somatic recordings), we were able to observe MCH axonal activation responses to both the food pellet and the cue in the mNAcSh, which were maintained across learning (Fig. S2A), and across a run. However, due to the lower signal quality of the mNAcSh terminal recordings, we focused on somatic recordings in subsequent experiments. B) lNAcc DA release dynamics in the early part of a run (first quintile of trials) vs the later part of a run (last quintile of trials) showing response to the cue (left) and acquisition of the food-reward (right). Inset:  Quantifications of tone and pellet responses (AUC of baseline corrected Response, Early vs. Late trials, within-run, n=6, two-tailed paired t-test, tone: N.S.. pellet: p<0.0635, t=2.3753, df=5). Pellet responses, but not tone responses, are attenuated over the course of a run (adaptation). Heatmaps are from a representative individual, showing one run (43 trials), aligned to tone (left) or pellet (right). C) As in A, but for mNAcSh DA release dynamics. Inset: (AUC of Response – Baseline, Early vs. Late trials, within-run, n=9, two-tailed paired t-test, tone N.S. pellet N.S). Adaptation of pellet responses in the mNAcSh is less apparent vs. the lNAcc. Heatmap from a representative individual. D) As in A, but for MCH neuron dynamics. Inset: (AUC of Response – Baseline, Early vs. Late trials, within-run, n=9, two-tailed paired t-test, tone N.S. pellet N.S.). Heatmap from a representative individual.


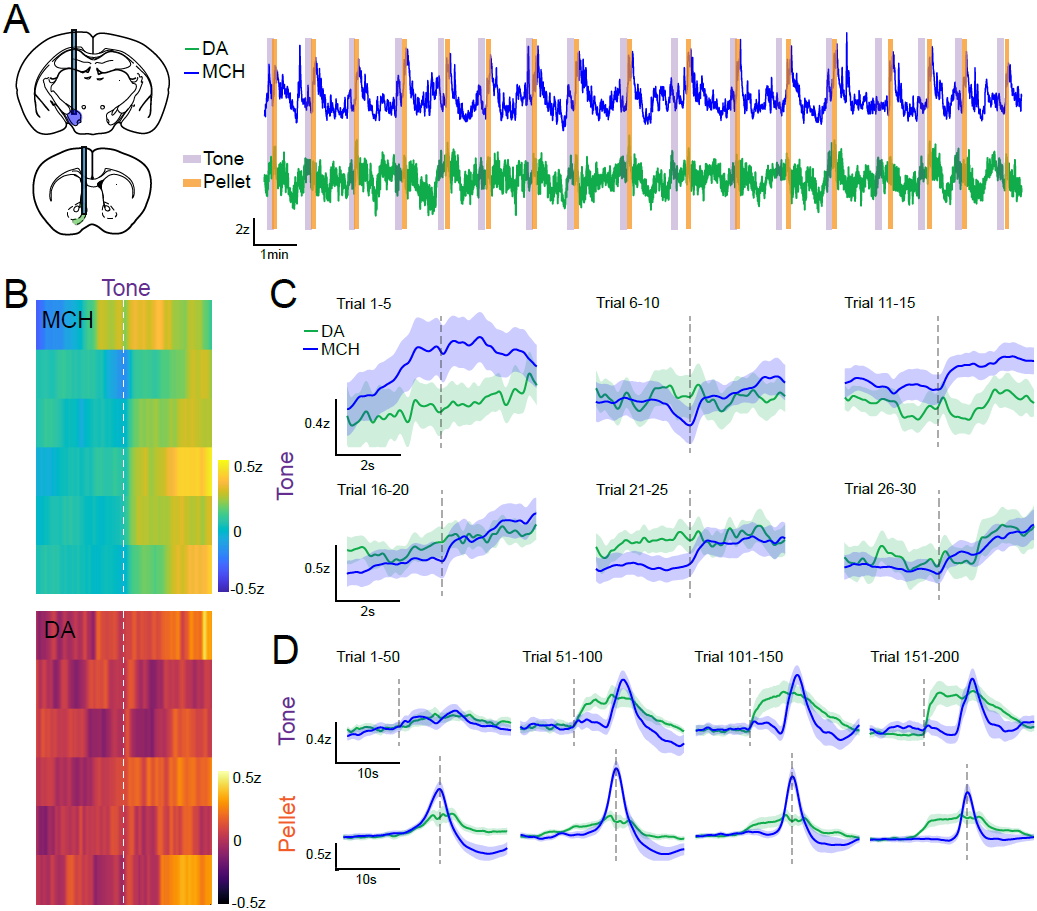


**Figure S6. Simultaneous measurement of mNAcSh DA release and LH/ZI MCH neuron dynamics across Pavlovian conditioning.**

A) Example simultaneous traces of MCH neuron dynamics (blue, top) and mNAcSh DA release (red, bottom) during Pavlovian conditioning, with tone/pellet presentations demarcated. B) (Top) Example individual heatmap showing cue-aligned MCH responses over the first   30 cue-reward pairing, after binning together every 5 consecutive trials (n=6, ‘responders’ only). (Bottom) Example individual heatmap showing cue-aligned DA responses over the first 30 cue-reward pairing, after binning together every 5 consecutive trials (n=6, ‘responders’ only). C) Across-animals (n=6) mean cue (tone)-aligned MCH and DA responses over the first 30 cue-reward pairings, after binning together every 5 consecutive trials. A consistent but small MCH neuron response to tone emerges very early, prior to the DA tone response. D) Across-animals (n=6) mean cue (tone)-aligned (top) or pellet-aligned (bottom) MCH/DA responses over the first 200 cue-reward pairings, after binning together every 50 consecutive trials. Although the DA response to tone is of much greater magnitude, it emerges later than the MCH tone response.


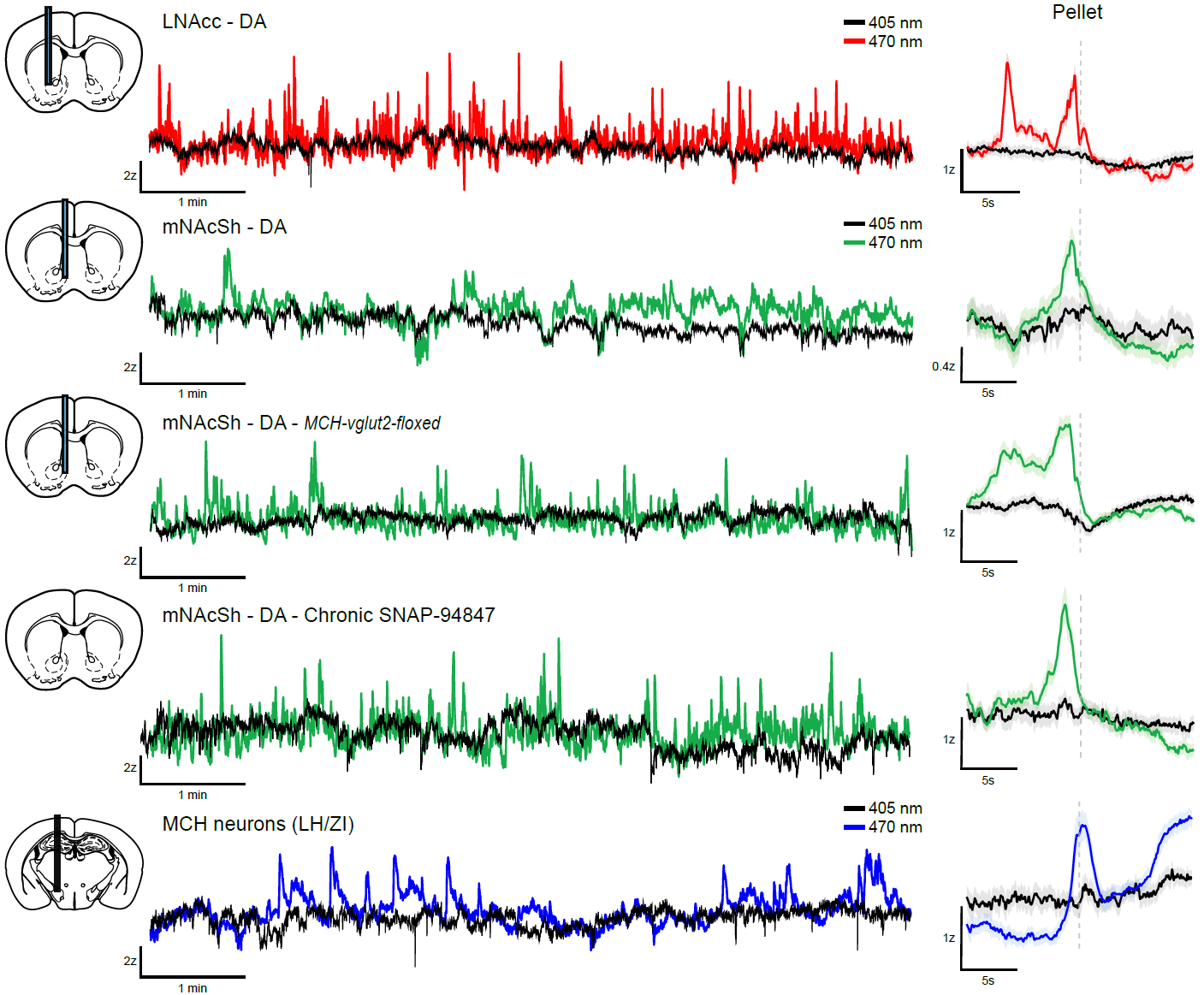


**Figure S7. Example 405nm (isosbestic) traces vs. 470nm (calcium-dependent) traces during Pavlovian conditioning for various sensors and fiber locations.**

Individual traces of 470nm (calcium-dependent) and simultaneous 405nm (isosbestic) fluorescence during Pavlovian conditioning from the various sensors, fiber locations, and experiments (Left). Individual mean (single-run) peri-event 470nm and 405nm traces (time locked to pellet retrieval) from each sensor and location (Right). Some small motion effects are visible, but not prominent in averaged data – however, we lacked isosbestic data for many mice/groups.


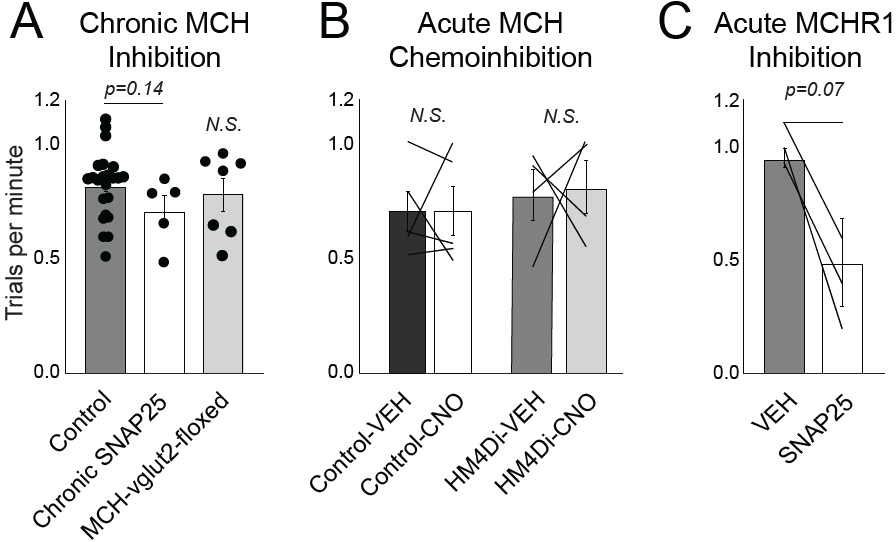


**Figure S8. Effects of MCHR1/MCH neuronal inhibition on response rates in the Pavlovian conditioning task.**

A) Across-animals mean trial-rate (trials per minute) in chronic MCH system-inhibited animals. (Two-tailed t-test vs. Control group, Control n=24, Chronic SNAP-94847 n=5, p=0.1371, t=1.532, df=27, MCH-vglut2-floxed n=5, N.S.) Author’s Note: Although comparisons of fluorescence magnitude between disparate groups of animals with fiber photometry can be fraught, we also looked for fluorescence differences between treatment groups. Neither chronic loss-of-function manipulation produced statistically significant alterations in mean pellet response magnitude at any time point compared to controls. B) Across-animals mean trial-rate in acute MCH chemo-inhibited animals (late learning). (2-Way RmANOVA VEH vs CNO-treated, Control vs MCH-HM4Di group, Control n=5, HM4Di n=4, All comparisons N.S.) C) Mean trial-rate in acute MCHR1 inhibited animals (late learning). (Two-tailed paired t-test vehicle treated (VEH) vs SNAP-94847 (25mg/kg I.P.) treated (SNAP25), n=4, p=0.0702, t=2.759, df=3).


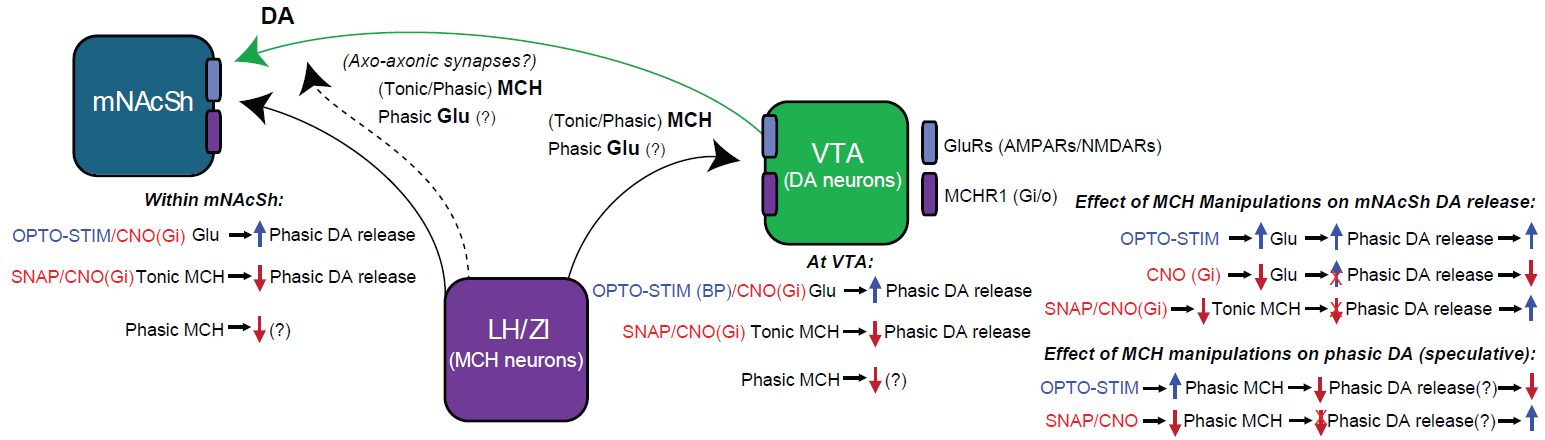


**Figure S9. Proposed circuit mechanisms underlying observed changes in DA release.**

Extended schematic of proposed circuit mechanisms, plus effects of manipulations on DA release (observed and speculated).

**Supplementary methods**

**Mice**

Mice were housed in a University of Michigan vivarium in a temperature-controlled environment (12 h light and 12 h dark cycle; lights on at 2 AM). Following recovery from surgery, mice were food restricted as described below, but maintained ad libitum access to water. Protocols were approved by the University of Michigan's Institutional Animal Care and Use Committee, and are in accordance with NIH guidelines for the use and care of Laboratory mice. MCH-Cre (Strain #: 014099) mice and littermate controls were used in these experiments. PmchΔVglut2 mice, Pmch-iCre crossed with Slc17a6tm1Lowl/J (Vglut2flox) mice (JAX® stock # 012898) were also used (ref. PMID: 17488640, PMID: 39007235).

**Histology**

We performed post-mortem histology on all animals to confirm fiber locations and construct expression (see Fig. S1 for examples). In one figure we made use of an anterograde tracer experiment from the Allen Brain Connectivity Atlas [20]. Eight images from the atlas were processed to remove background and thresholded prior to being quantified. We also quantified striatal MCH-ChrimsonR+ terminals in mice we generated (see optogenetics experiments for details on how mice were generated). 1-7 histology images from each of 3 mice were processed and quantified as above.

**Surgery**

Mice were deeply anesthetized by inhalation of 2% isoflurane and placed on a stereotaxic apparatus (Tujunga, CA). Following standard disinfection procedure, a small hole was drilled into the skull unilaterally at defined positions to target lNAcc (A/P: +1.2mm, M/L: -1.3mm, D/V: -4.1mm), mNAcSh  (A/P: +1.1mm, M/L: -0.53mm, D/V: -4.6mm), LH/ZI  (A/P: -1.6mm, M/L: +0.9mm, D/V: -4.8mm relative to bregma). A pulled-glass pipette was inserted into the brain, and virus was injected by picospritzer used to control injection speed at 25 nl/min. For fiber photometry experiments, 200 µL of AAV5-CAG-dLight1.1 (AddGene #111067-AAV5; titer 7 x 10^12^ genome copies per mL), AAV9-hSyn-GRAB-rDA1m (AddGene #140556-AAV9; titer 1 x 10^13^ genome copies per mL), or 500 µL bilaterally of AAV1.Syn.Flex.GCaMP6s.WPRE.SV40 (AddGene #100845-AAV1;  titer 1 x 10^13^ genome copies per mL) or pGP-AAV-hSyn-FLEX-jGCaMP7s-WPRE (AddGene #104441-AAV9; titer 1 x 10^13^ genome copies per mL) was injected into the region of interest (Note: GCaMP mice were pooled regardless of virus used). For chemogenetic and optogenetic experiments, 500 µL of AAV5-hSyn-DIO-HM4Di(Gi)-mcherry (AddGene #44362-AAV5; titer 7 x 10^12^ genome copies per mL) or AAV5-Syn-FLEX-rc[ChrimsonR-tdTomato] (AddGene #6723-AAV5; titer 5 x 10^12^ genome copies per mL) were injected bilaterally in the LH region of pMCH-Cre mice, as well as AAV5-CAG-dLight1.1 in the striatum. An optic fiber (400-µm diameter core; BFH37-400 Multimode; NA 0.37 or 0.5; ThorLabs) was implanted over the region(s) of interest. After surgeries, mice were housed individually and monitored for proper recovery.

**In vivo fiber photometry**

For behavioral experiments mice were food restricted to ~85% body weight. Beginning 3 weeks after surgery, mice were connected to a fiber optic patch cable. Fiber optic patch cables (400 μm diameter; Doric Lenses) were firmly attached to the implanted fiber optic cannulae (Doric Lenses). LEDs (Plexon; 473 nm, Thorlabs; 405nm, Doric; 560nm) were set such that a light intensity of <0.2mW entered the brain; light intensity was kept constant across sessions for each mouse. Emission light was passed through a filter cube (Doric) before being focused onto a sensitive photodetector (Newport 2151 or Doric 119222-06). Signals were digitized at 1000 Hz using a National Instrument data acquisition system (or resampled up to 1000 Hz from 130 Hz in some experiments). In some experiments where 405 nm isosbestic signals were simultaneously acquired, the 405 nm excitation was either interleaved (time-domain) with the 473 nm excitation at 130Hz, or interleaved (frequency domain) at 217Hz or 319 Hz (10kHz recording). Multiplexed data was subsequently demodulated using custom MATLAB scripts. The signal was corrected by subtracting a double exponential fit, then adding back the mean of the trace. For each recording session, the signal was converted to ΔF/F ((F – F_0_)/F_0_); where F_0_ was calculated as the 10th percentile of the entire fluorescence trace) and subsequently normalized as a z-scored ΔF/F.

**Feeding experiments**

Feeding experiments were performed in the last 2 hours of the light cycle or first 2 hours of the dark cycle. Each session consisted of 4-10 alternating food and bedding drops. Only one session was run per day and mice underwent 1-2 sessions. All trials across days were pooled to calculate the response to food/bedding in each animal. In ‘free feeding’ experiments, photometry and video recordings were initiated, and mice were placed in a fresh cage. After habituation to the cage and establish baseline photometry levels, several pieces of regular chow were placed into one corner of the cage after ~30s, this period was included in the main figure analysis (Fig. 1D-J) but excluded in the corresponding supplemental figure (Fig. S3A-D). Mice were then allowed to interact with the food for 20-30 minutes. For subsequent analyses, photometry data was synchronized with the video recording, and a human scorer noted the times during which the mouse consumed the chow.

**Pavlovian conditioning**

Mice had two 30 min sessions to habituate to the recording chamber, in-cage FED3 [22], and optic cable and were allowed to freely receive pellets. Mice were trained across 3-11 subsequent sessions to associate a 1 kHz, 5s-duration tone with pellet delivery. ~40 tones were played per session, with pellet delivery occurring 5 s after tone onset, and a variable 60-90s ITI. Both the start of the tone and the moment of pellet retrieval were timestamped via TTL pulses.

**Gain and loss of function experiments**

For DREADD experiments, MCH-Cre mice were injected with either AAV5-hSyn-DIO-HM4Di(Gi)-mcherry or AAV5-Syn-FLEX-rc[ChrimsonR-tdTomato] (for controls) in the LH/ZI and dLight1.1 (mNAcSh) during fiber implantation surgery. A minimum of 5 weeks was allowed for viral expression before experiments. Mice were run through the Pavlovian conditioning paradigm as described elsewhere in methods, except that (30 min.) prior to the start of 1-2 runs, mice were habituated to I.P. injections of saline. After mice were fully trained, they proceeded to the 2 DREADD-experimental days. On the first experimental day, mice were pretreated with vehicle (0.5% DMSO in 0.9% saline, I.P.) 30 min. prior to running in the Pavlovian task as before. On the 2^nd^ experimental day, mice were pretreated with 3mg/kg CNO (I.P.) dissolved in vehicle 30 min. prior to the task.

For optogenetics experiments, MCH-Cre mice were injected with AAV5-Syn-FLEX-rc[ChrimsonR-tdTomato] (in LH/ZI) and dLight1.1 (mNAcSh), and implanted with mNAcSh recording fibers. As elsewhere, mice had time to recover and undergo normal food-restriction and habituation. As in the DREADD experiments, mice were already trained on the Pavlovian conditioning paradigm prior to the onset of the optogenetic experiments. The optogenetic stimulation paradigm was identical to the normal Pavlovian conditioning paradigm, except that in a proportion of trials (70% :30% stim:no stim) either the tone, the pellet, or both (~10% of stim trials) were paired with optogenetic stimulation (5s-duration, 20Hz, 10ms pulse-width, 625 nm). Stimulation was delivered through the mNAcSh fiber, and was calibrated to approximately 9mW at the fiber tip using a portable light meter. Optogenetic stimulation was triggered by a custom Arduino script and was recorded with TTL pulses by the DAQ for subsequent analysis. Removal of stimulation artifacts proved unnecessary in these mice due to the frequency modulation of 473/405nm excitation light.

**Pharmacology**

For the chronic MCHR1 inhibition experiment, dLight1.1-expressing mice were run through the Pavlovian conditioning paradigm as described; however, 45 min. prior to the start of each run, mice were treated with SNAP-94847 (25 mg/kg, I.P., dissolved in 20% w/v β-cyclodextrin/saline). Mice were habituated to saline injections prior to the experiment. This dose was chosen as a similar dose (30 mg/kg I.P.) has previously been reported to have behavioral effects and result in high MCHR1-occupancy (estimate 80%+) [33,34] in mice, and appeared to produce less sedation/behavioral inhibition compared to 30 mg/kg.

The acute MCHR1 inhibition experiment was conducted as above, except mice had been previously trained without pharmacology in the Pavlovian conditioning paradigm. As in the DREADD experiments, mice were initially pretreated with vehicle (20% w/v β-cyclodextrin/saline) on the first of 2 days. On the 2^nd^ experimental day, mice were pretreated with SNAP-94847 (25 mg/kg, I.P).

**Immunohistochemistry**

Following the conclusion of all experiments, mice were anesthetized with pentobarbital and transcardially perfused with phosphate-buffered saline (PBS) followed by formalin. Once removed, brains were post-fixed in 10% formalin overnight then transferred to 20% PBS-sucrose for 48 hours. Brains were then frozen and 40μm coronal sections were cut using a freezing microtome. Fluorescent images were captured with a Keyence BZ-X810 slide scanner microscope.

**2-color photometry analyses**

Analysis was performed as in [23], using MATLAB scripts adapted from their work. Briefly, after normal signal processing, synchronously recorded GRAB-rDA1m and GCaMP were analyzed for cross-correlation (using MATLAB – xcorr function), coherence and phase-offset in the 0-10Hz range. Coherograms used a multi-taper estimation with the chronux function cohgramc (window, 10 s; overlap, 5 s; step, 5; padding, 0). Feeding/Pavolvian epochs were expanded by 3s prior to their start and 5s at the end to catch transitions. To generate the phase-offset curves, we likewise adapted the analysis in [23]– briefly, the signals were processed with a Butterworth (bandpass) filter (Order 4, Range 1-3Hz or 3-7Hz) and the phase angle of the Hilbert function (MATLAB - hilbert) was extracted. Photometry fluorescence amplitudes were then extracted and averaged in 10 degree bins from -180 to +180 degrees. The resultant curves were normalized between 0 and 1 across the full oscillatory cycle and superimposed for the figures. Curves were smoothed with a moving average (2s - cross correlation, 0.6Hz - coherence/phase offset, or 60 degrees - offset curves) for figures to improve interpretability.

**Statistical analysis**

Statistical analyses were performed using Prism 9.0 (GraphPad) or Matlab software. Data presented met the assumptions of the statistical test employed. Exclusion criteria for experimental mice were (i) sickness or death during the testing period (ii) if histological validation of the injection site demonstrated an absence of reporter gene expression (iii) or if histological validation demonstrated a mistargeted fiber placement. These criteria were established before data collection. N numbers represent final numbers of healthy/validated mice, except in the case of one mouse where histology was unavailable.
